# Neoadjuvant radiochemotherapy in the treatment of fixed and semi-fixed rectal tumors. Analysis of results and prognostic factors

**DOI:** 10.1186/1748-717X-1-5

**Published:** 2006-03-28

**Authors:** Robson Ferrigno, Paulo Eduardo Ribeiro dos Santos Novaes, Maria Letícia Gobo Silva, Ines Nobuko Nishimoto, Wilson Toshihiko Nakagawa, Benedito Mauro Rossi, Fábio de Oliveira Ferreira, Ademar Lopes

**Affiliations:** 1 Department of Radiation Oncology, Hospital do Câncer A. C. Camargo, Rua Prof. Antonio Prudente, 211, São Paulo, SP 01509-900, Brazil; 2 Department of Biostatistics, Fundação Antonio Prudente, Rua Prof. Antonio Prudente, 211, São Paulo, SP 01509-900, Brazil; 3 Department of Pelvic Surgery, Hospital do Câncer A. C. Camargo, Rua Prof. Antonio Prudente, 211, São Paulo, SP 01509-900, Brazil

## Abstract

**Purpose:**

To report the retrospective analysis of patients with locally advanced rectal cancer treated with neodjuvant radiochemotherapy.

**Methods and Materials:**

From January 1994 to December 2003, 101 patients with fixed (25%) or semi-fixed (75%) rectal adenocarcinoma were treated by preoperative radiotherapy with a dose of 45Gy at the whole pelvis and 50.4Gy at primary tumor, concomitant to four weekly chemotherapies with 5-Fluorouracil (425 mg/m^2^) and Leucovorin (20 mg/m^2^). In 71 patients (70.3%) the primary tumor was located up to 6 cm from the anal verge and in 30 (29.7%) from 6.5 cm to 10 cm. Age, gender, tumor fixation, tumor distance from the anal verge, clinical response, surgical technique, and postoperative TNM stage were the prognostic factors analyzed for overall survival (OS), disease-free survival (DFS), and local control (LC) at five years.

**Results:**

Median follow-up time was 38 months (range, 2–141). Complete response was observed in eight patients (7.9%), partial in 54 (53.4%) and absence in 39 (38.7%). OS, DFS and LC were 52.6%, 53.8%, and 75.9%, respectively. Distant metastasis occurred in 40 (39.6%) patients, local recurrence in 20 (19.8%) and both in 16 (15.8%). Patients with fixed tumors had lower OS (17% Vs 65.6%; p < 0.001), DFS (31.2% Vs 60.9%; *p *= 0.005), and LC (58% Vs 82%; *p *= 0.004). Patients with tumors more than 6 cm above the anal verge had better LC (93% Vs 69%; *p *= 0.04). The postoperative TNM stage was a significant factor for DFS (I:64.1%, II:69.6%, III:35.2%, IV:11.1%; *p *< 0.001) and for LC (I:75.7%, II: 92.9%, III:54.1%, IV:100%; *p *= 0.005). Patients with positive lymph nodes had worse OS (37.9% Vs 70.4%, *p *= 0.006), DFS (32% Vs 72.7%, *p *< 0.001) and LC (56.2% Vs 93.4%; *p *< 0.001).

**Conclusion:**

This study suggests that the neoadjuvant treatment employed was effective for local control. Fixation of the lesion and lymph nodes metastasis were the main adverse prognostic factors. Distant failures were frequent, supporting the need of new drugs for adjuvant chemotherapy.

## Introduction

The employment of preoperative radiotherapy (RT) combined or not with chemotherapy (CT) has been used in the treatment of rectal cancer for the past two decades and its employ gradually increased as adjuvant therapy, especially in T3/T4 and/or N1/N2 tumors [1,2]. The strategy of performing preoperative instead of postoperative treatment, has the proven advantages of lower acute toxicity [3-6], lower total dose of radiation needed [4] and eventual tumor regression and downstaging to enable curative resection and even sphincter preservation [7-17]. Furthermore, some authors showed better local control with preoperative RT when compared to surgery alone [7-10,18,19]. Upon comparison with the postoperative radiochemotherapy approach for adjuvant treatment, data suggest that local control was better using preoperative radiochemotherapy [20]. In preoperative therapy, the association of CT increases pathologic downstaging when compared to radiation alone [21]. Theoretical advantages of the preoperative strategy include increased radiosensitivity due to more oxygenated cells and decrease of tumor seeding during surgery [22]. For patients with fixed or tethered tumors to adjacent structures, the goal of preoperative RT, preferably combined with CT, is to achieve maximal tumor regression to facilitate resection.

This study reports results on patients with fixed and semi-fixed adenocarcinoma of the rectum treated with preoperative radiochemotherapy, as well as the analysis of some prognostic factors that could have influenced the outcome.

## Methods and materials

### Patient and tumor characteristics

From January 1994 to December 2003, 101 patients with locally advanced rectal cancer, characterized by fixed or semi-fixed tumor, were treated with preoperative RT concomitant to CT. All patients had biopsy proven adenocarcinoma of the rectum and they were staged through physical exam, including digital rectal examination of the primary lesion by the same team of surgeons, chest radiograph, computerized tomography of the abdomen and pelvis, blood chemistries, HIV test and colonoscopy. Endorectal ultrasound was not used for staging these patients. A semi-fixed tumor was that with preserved mobility in at least one direction at digital rectal examination. The tumor distance from the anal verge was measured by colonoscopy. Table 1 summarizes the patients and tumor characteristics.

**Table 1:** Patients and tumor characteristics.

Patient number	101
Period	Jan/1994 – Dec/2003
Age (year)	
Median	62
Range	(25 – 84)
Gender	
Male	52 (51.5%)
Female	49 (48.5%)
Tumor distance from the anal verge	
0–6 cm	71 (70.3%)
6.5–10 cm	30 (29.7%)
Tumor mobility	
Fixed	25 (24.7%)
Semi-fixed	76 (75.3%)

### Radiotherapy

All patients received whole pelvic radiation with dose of 45Gy in 25 daily fractions of 1.8Gy, over five weeks, by four fields, followed by a boost to the primary tumor of up to 50.4Gy, with at least 2 cm margins, by three fields (one posterior and two laterals). The upper limit of all the pelvic fields was at the L5-S1 level and the lower one was 4 to 5 cm below the tumor. The lateral fields covered the sacrum and coccyx posteriorly and the femoral head anteriorly. The photon energy used was given by a 4 or 6 MV linear accelerator. The dose was prescribed to the 95% isodose line. All fields were treated daily and weighting was 2:1 for the posterior – anterior and laterals incidences, respectively, for four fields whole pelvis, and 2:1:1 for the posterior, right lateral, and left lateral portals, respectively, for three fields boost. Wedges of different degrees were employed over the lateral fields to homogenize the isodose distribution. The isodoses distribution was designed by 2D treatment planning system.

### Chemotherapy

The CT was performed with two hours bolus infusion of 5-Fluorouracil (5-FU) and leucovorin (LV), once a week, with a median of four cycles (range: 2-6). The median dose of 5-FU per cycle was of 425 mg/m^2 ^(range: 88 – 800 mg/m^2^) and all patients treated with CT received 20 mg/m^2 ^of LV. During the radiochemotherapy course, acute toxicity was evaluated. If nausea, vomiting, diarrhea, mucositis or leucopenia were not controlled with medication, the treatment was temporarily interrupted. The decision of performing this weekly CT schedule instead of during the first and last week of RT course had the objective of maximize the radiation effect.

Adjuvant CT was employed in all patients with postoperative lymph-nodes metastasis and in those who presented unresectable primary tumor or intrabdominal disease dissemination during surgery. This CT was based on 5-FU and LV.

### Preoperative evaluation and surgery

Four weeks after the radiochemotherapy course, all patients were evaluated and restaged by means of physical examination, computerized tomography of the abdomen and pelvis, chest x-ray, blood chemistries, and colonoscopy. If at colonoscopy no tumor was visualized, patients were considered as having a complete clinical response, partial response was considered if tumor regressed more than 50% of the initial volume, and no response if the tumor did not regress more than 50%. Surgery was planned to take place four to six weeks after the radiochemotherapy course. The surgical technique was decided by the surgeon's team, based on tumor location, clinical response, and intraoperative findings. All patients treated with surgery underwent total mesorectal excision by means of anterior resection, abdominoperineal resection or pelvic exenteration. Postoperative stage was classified by the American Joint Committee on Cancer (AJCC) TNM staging system [23], based on pathologic findings. Patients with complete pathologic response were considered as stage 0 (T0N0M0).

### Follow-up

Follow-up was performed at every 3 months in the first two years following completion of surgery, and at a minimum of 6 months thereafter. At each follow-up all patients underwent clinical examination and also a rectosigmoidoscopy in those treated with sphincter saving surgery. Chest radiograph and abdominopelvic computerized tomography were done every 6 months in the first 3 years and every 12 months thereafter or when clinically required.

### Statistical analysis

All statistical analyses were performed with a software program Statistics/Data analysis (STATA Corporation, Houston: University of Texas; 2000). Overall survival (OS), disease free survival (DFS), and local control (LC) were calculated according to the actuarial method of Kaplan and Meier [24]. The calculation of OS, DFS and LC was performed from the date of diagnosis to the date of the event. Survival was measured from the date of diagnosis to death or last follow-up. Patients who died of diseases unrelated to cancer were censored. The prognostic factors analyzed were: patient's age, gender, pretreatment tumor status (fixation), tumor distance from the anal verge, clinical response to the neoadjuvant treatment by colonoscopy, surgical technique employed, and postoperative TNM stage. The log-rank test was used to compare the actuarial probabilities curves for OS, DFS and LC. Relative risk of death was determined by Cox regression analysis [25]. Comparison of categorical variables was performed using the chi-square (χ^2^) test. Values of *p *lesser than 0.05 or 95% were considered as having a statistical significance. Last revision of this analysis was carried out in July 2005.

## Results

### Neadjuvant treatment

Of the 101 patients treated, 7 (6.9%) did not complete the prescribed dose of preoperative RT because of persistent neutropenia and/or diarrhea. Of these, two died due to septicemia and the other five underwent surgery before the end of radiochemotherapy. Doses administered to these patients ranged from 14.4Gy to 39.6Gy at the whole pelvis. During the RT course, 88 (87.1%) patients received concomitant weekly CT. Thirteen patients (12.9%) did not receive CT because of inadequate clinical conditions. Temporary interruption of both treatment (RT and CT) with a median duration of one week, due to leucopenia, diarrhea or mucositis not controlled with medication, was necessary in 22 (21.8%) patients (grade 3 toxicity). The rate of treatment response, evaluated four weeks after the end of RT, was considered complete in eight (7.9%) patients, partial in 54 (53.4%), and null in 39 (38.6%). None of the patients developed tumor progression during or up to four weeks after RT.

### Surgery

Surgery was performed four to six weeks after RT in 89 patients (88%). Of these, 83 (82%) had the primary tumor removed and 6 (5.9%) underwent only colostomy because of unresectable tumor and/or disease dissemination detected during laparotomy. All patients treated by surgery underwent total mesorectal excision and according to the surgical technique employed for tumor removal, 38 (37.6%) were by anterior resection (AR), 36 (35.6%) were by abdominoperineal resection (APR), and 9 (9%) were by pelvic exenteration. All 83 patients with surgical removal of the tumor had negative resection margins, including the circumferential one.

Twelve (11.8%) patients were not submitted to surgery because two died during the neoadjuvant treatment, five presented distant metastasis at restaging procedures and five refused surgery because they achieved complete clinical response after radiochemotherapy course. These last patients have been followed up every three months. One developed distant metastasis after 14 months of follow-up and died 17 months after diagnosis with no local failure. This patient was initially staged as T4 because of vaginal invasion. The other four patients are alive with no evidence of disease with median follow-up of 72 months (range: 48 – 96). These patients had the primary tumor located from 2 to 6 cm from the anal verge and they were considered candidates to APR by the surgeon prior to neoadjuvant treatment.

### Sphincter preservation

Among 71 patients with distal rectal cancer (tumor up to 6 cm from the anal verge) and initially considered candidates to APR, 14 (19.7%) underwent sphincter-sparing low AR and coloanal anastomosis. Of these, one patient had tumor located 2 cm from the anal verge, one had it located 3 cm away and the remaining at a 4 to 5 cm distance. In this group, the 5-year local control probability was of 58.8%. The patient with the tumor 2 cm from the anal verge was postoperative stage T3N2M0 and developed both local and distant failures. Adding these patients with the five with distal rectal cancer who refused surgery and did not develop local failure, the sphincter preservation rate among patients with initial indication of APR was of 26.8% (19/71).

### Postoperative staging and surgical findings

The postoperative TNM staging of the 83 patients with primary tumor removed by surgery, according to AJCC is shown in Table 2. The primary tumor was not removed in six patients because it was unresectable in three, two had intrabdominal disease dissemination and one presented both. Among patients with the primary tumor removed by surgery, 33 (39.7%) had lymph-node metastasis at pathology report (N1/N2) and their T stage distribution was: T0:2 (2.4%); T1:2 (2.4%); T2:26 (31.3%); T3:42 (41.6%); and T4:11 (13.2%). Of the 25 patients with initially fixed tumors, 16 (64%) underwent tumor resection by AR (5 patients), APR (6 patients) or pelvic exenteration (5 patients).

**Table 2:** Postoperative TNM staging distribution by AJCC

Stage	n (%)
0	2 (2.4%)
T0N0M0	2 (2.4%)
I	20 (24.1%)
T1N0M0	2 (2.4%)
T2N0M0	18 (21.7%)
II	26 (31.3%)
T3N0M0	22 (26.5%)
T4N0M0	4 (4.8%)
III	30 (36.1%)
T2N1-2M0	7 (8.4%)
T3N1-2M0	16 (19.3%)
T4N1-2M0	7 (8.4%)
IV	5 (6%)
T2N1M1	1 (1.2%)
T3N0M1	2 (2.4%)
T3N1M1	2 (2.4%)

*Abbreviation: *AJCC = American Joint Committee on Cancer.

### Patient's follow-up and patterns of failure

Median follow- up time was 38 months (range, 2 – 141). At the time of this analysis, 46 patients (45.5%) were alive with no evidence of disease, 5 (4.9%) were alive with evidence of disease, 42 (41.6%) died due to the rectal cancer, 4 (4%) died of second primary tumor, and 4 (4%) died due of diseases unrelated to cancer. According to the pattern of failure, 24 (23.8%) patients developed only distant metastasis, 4 (4%) had only local recurrence, and 16 (15.8%) developed both. Two patients who developed only local failure were rescued by a second surgery. Eight (7.9%) patients developed second primary tumor. Of these, two had lung cancer and died; one had low grade non Hodgkin's lymphoma and is alive with no evidence of disease; one had bladder cancer and died of causes unrelated to cancer; one had kidney cancer and died of rectal cancer; one developed prostate cancer and is alive with no evidence of disease; one developed acute lymphoblastic leukemia and died of it, and one died due to a glioblastoma multiform of the brain.

### Actuarial results and prognostic factors

**Figure 1:**
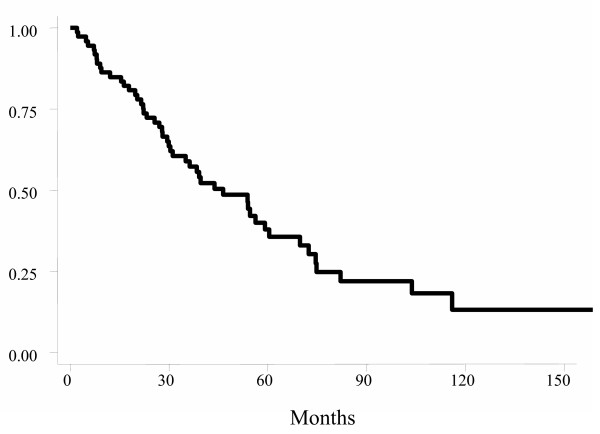
Actuarial overall survival probability for all patients.

**Figure 2:**
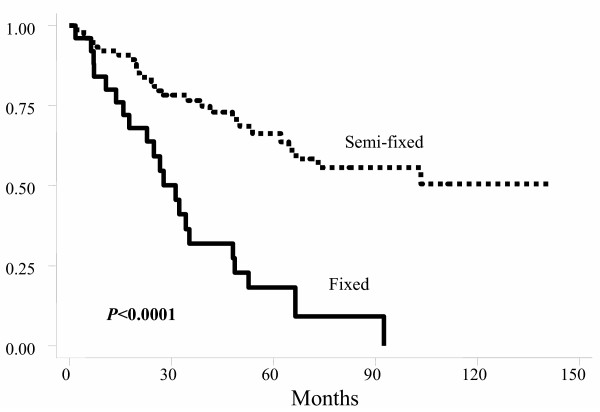
Actuarial overall survival probability by tumor fixation.

**Figure 3:**
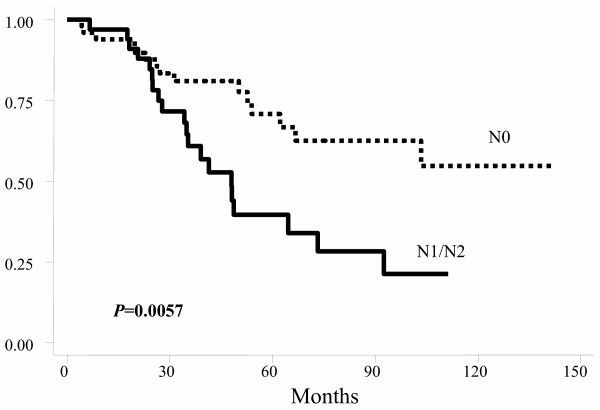
Actuarial overall survival probability by postoperative lymph-nodes stage.

Using the Kaplan-Meier actuarial method, probabilities of OS, DFS, and LC at five years for all patients were 52.6% (Figure 1), 53.8%, and 75.9%, respectively. For OS, age, gender, tumor location, postoperative TNM stage, and clinical response were not statistically significant factors. Patients with fixed tumor had worse 5-year OS (17% Vs 65.7%; *p *< 0.001) (Figure 2) as well as those with positive postoperative lymph nodes (37.9% Vs 70.4%; *p *= 0.006) (Figure 3).

**Figure 4:**
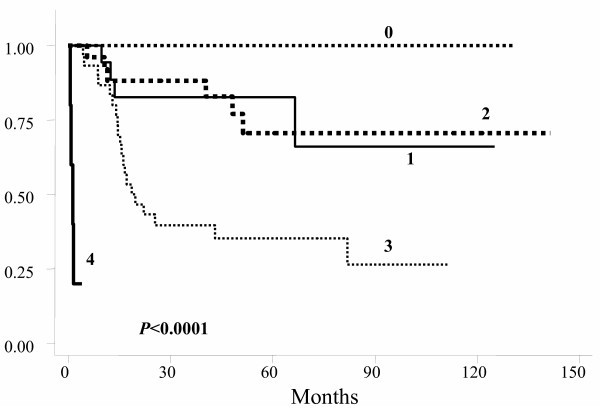
Actuarial disease-free survival probability by postoperative TNM stage.

For DFS, age, gender, tumor location, surgical technique, and clinical response were not statistically significant factors. Patients with fixed tumors had worse DFS, as well as those with positive postoperative lymph-nodes (Table 3), and those with postoperative stages III and IV (Table 3 and figure 4).

**Table 3:** Disease-free survival (DFS) and local control (LC) probability at five years by prognostic factors.

Prognostic factor	Category	DFS	*P*	LC	*p*
Tumor fixation	Semi-fixed	60.1%	0.005	81.9%	0.004
	Fixed	31.2%		58.2%	
Tumor distance from the anal verge	≤ 6 cm	48.1%	0.19	69.3%	0.043
	> 6 cm	67.1%		92.6%	
Postoperative stage	I	64.1%	<0.001	75.7%	0.005
	II	69.6%		92.9%	
	III	35.2%		54.1%	
	IV	11.1%		100%	
Postoperative N stage	N0	72.7%	<0.001	93.4%	<0.001
	N1/N2	32.0%		56.3%	

**Figure 5:**
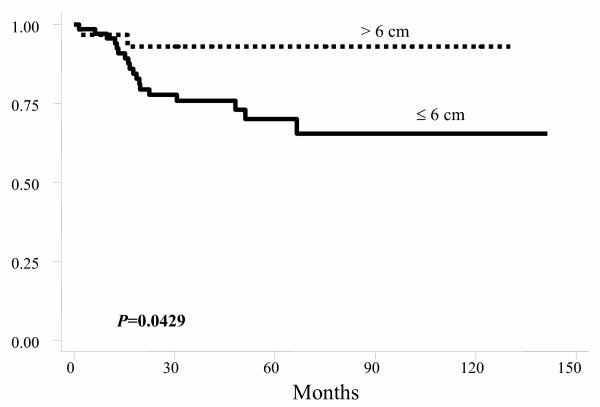
Actuarial local control probability by tumor distance from the anal verge.

The probability of LC at five years was not influenced by age, gender, clinical response, surgical technique, and postoperative T stage. Better 5-year LC was observed in patients with semi-fixed tumor (Table 3) and in those with tumor located above 6 cm from the anal verge (Table 3 and figure 5). Patients with postoperative stage III disease had lower 5-year local control, as well those with postoperative positive lymph-nodes (Table 3).

Estimated relative risk of death, calculated by Cox regression analysis, was higher among patients with fixed tumors and with postoperative positive lymph-nodes (Table 4).

**Table 4:** Death risk according to the main prognostic factors by Cox multivariate regression analysis.

Variable	Category	HR*	[95% Conf. Interv.]	HR^§^	[95% Conf. Interv.]
Tumor fixation	Semi-fixed	1.0	Reference	1.0	Reference
	Fixed	3.87	(2.1 – 7.0)	2.64	(1.2 – 5.7)
Postoperative	N0	1.0	Reference	1.0	Reference
N stage	N1/N2	2.51	(1.3 – 4.9)	2.13	(1.0 – 4.4)

* Crude harzard risk

^§ ^Adjusted harzard risk for age (median of 61 years)

## Discussion

For locally advanced rectal cancer, the employment of preoperative radiotherapy, preferably combined with chemotherapy, is an interesting treatment strategy due to the possibility of tumor downstaging, which leads to an enhanced resectability rate [11,12,14-17]. Other advantages of this treatment strategy, already reported in literature, include sterilization of the tumor bed, easier displacement of the small bowel and a lower total dose of radiation needed [5,26-29].

In this study, all patients had locally advanced primary tumors and were classified as fixed or semi-fixed ones. After the radiochemotherapy course, 83 (82%) patients had the tumor resected with negative margins. The downstaging achieved was not precisely determined because the endorectal ultrasound was not used for staging, but 30 (29.7%) patients presented postoperative T0 – T2 tumors (Table 2). Furthermore, among 25 patients with fixed tumor and initially supposed to be unresectable, 16 (64%) was resected with negative margins. The actuarial 5-year local control rate of 75.9% was reasonable, considering the initial extension of the primary tumor. This result is consistent with other series from literature that report similar local control rates in patients with locally advanced tumors treated with preoperative radiotherapy with or without chemotherapy [12,15,16,30-37]. In our series, distant metastasis was the predominant pattern of failure. This implies the need of new drugs for adjuvant treatment for these patients.

For resectable rectal tumors, preoperative RT seems to achieve better local control than the postoperative RT, as reported by the prospective and randomized German trial CAO/ARO/AIO 94 [20] and by two metanalyses [38,39]. At our Institution, we still do not use preoperative RT for resectable tumors. This group of patients is at first treated with surgical resection and the indication of adjuvant treatment is determined by the pathology report. In the future, we will probably design a prospective and randomized phase III trial similar to the German one to compare preoperative with postoperative radiochemotherapy in the management of rectal cancer, even for resectable tumors.

Endorectal sonography can be useful for staging primary rectal tumors before surgery or preoperative radiochemotherapy, mainly for resectable tumors, which will help to elect the surgical technique. Although the accuracy of this exam is of approximately 70% [40,41], two recent studies with preoperative radiotherapy showed that it is insufficient to stage lymph-node involvement [42,43]. As all patients in our study at digital rectal examination had fixed or semi-fixed tumors, we did not use this exam during the staging procedures. Furthermore, postoperative pathology staging has been demonstrated to be a more accurate prognostic factor than the ultrasound staging [42-44].

Our Institution had already begun a prospective trial to test the possibility of avoiding surgery in patients with distal rectal adenocarcinoma who had presented pathologic complete response after 4 weeks of 50.4Gy of radiation at the whole pelvis, concomitant to CT with 5-FU (425 mg/m^2^/day) and LV (20 mg/m2/day) during the first 3 days and the last 3 days of RT [45]. These patients had received a radiotherapy boost at the primary tumor site with a 20Gy dose to replace surgical resection. Of 52 patients enrolled in this study, 10 (19.2%) achieved pathologic complete response and underwent radiation boost with no surgery. Of these, eight (80%) developed local recurrence within 3.7 to 8.8 months [46]. These findings have influenced our surgical team not to try sphincter preservation in distal rectal cancer, even after complete response to the radiochemotherapy course. Although sphincter preservation was not the main goal of our study, 19.7% patients initially candidate to APR underwent sphincter-sparing low AR and coloanal anastomosis. The 5-year local control in this group of patients was of 58.8%, suggesting that this strategy can compromise the local control.

In the management of distal rectal cancer, sphincter-sparing surgery is nowadays the main subject of controversy. One of the most important controversies is whether the degree of downstaging warrants this type of surgery [1]. Results of the German (CAO/ARO/AIO 94) randomized trial of preoperative versus postoperative combined radiochemotherapy suggest that this assessment is accurate [20]. A preliminary report of the NSABP R-03 trial revealed that the proportion of patients who underwent sphincter-sparing surgery and were disease free was higher in the preoperative than the postoperative arm (44% Vs 34%) and that the rate of sphincter preservation among distal rectal cancer patients was 23% [47], similar to our results. Unfortunately, this trial was closed early because of small patient accrual. Other series from literature report the rate of sphincter preservation among patients with initially resectable distal rectal cancer ranging from 30% to 70%, with local failure of approximately 10% [15,16,47-56]. At our Institution, we believe that more prospective trials with longer follow-up are required to authorize a change of philosophy about margin resection.

Curiously, five patients in our study refused surgical resection after complete clinical response. Of these, four are still alive with no evidence of disease with a relative long follow-up (48 – 96 months). Probably, in this group of patients, tumors had some molecular markers which afforded them better response to preoperative therapy. Some authors have already studied selected molecular markers such as c-K-ras, thymidylate synthase, p53, p27Kkip1, DCC, EGFR, TP53, Ki-67, and apoptosis to identify this group of patients [57-64]. However, these studies are still limited and in the future, it will be imperative to identify some groups of patients by means of tissue collections to better choose the most appropriate therapy, including treatment with no surgery. Currently, observation is still not recommended for clinical complete responders. This affirmation is supported by the retrospective analysis of 488 patients with rectal cancer from the Memorial Sloan-Kettering Cancer Center treated with preoperative radiochemtoherapy. The clinical complete response rate was 19% and of these, pathologic complete response was observed in only 25%, showing that a significant percentage of clinical complete responders had persistent deep tumors or nodal involvement. The authors concluded that all patients with rectal cancer should undergo resection, regardless of their response to preoperative therapy [65]. Furthermore, locoregional tumor control should not be jeopardized by the justification of quality of life (QOL). Two recent analyses about QOL among patients with rectal cancer treated by preoperative radiotherapy showed that the presence of a permanent stoma did not affect the QOL outcome, when compared with patients treated with sphincter-sparing surgery [66,67].

In our study, the main adverse prognostic factors were fixation of the primary tumor and the presence of lymph-node metastasis (Table 3 and figure 2). Postoperative TNM stage was a prognostic indicator for disease-free survival and local control but not for overall survival (Table 3). These findings reflect the influence of tumor extension at the time of diagnosis, which can lead to distant dissemination, the main cause of death among our patients. Other series from literature also describe the postoperative TNM staging as a strong prognostic factor, especially if pelvic lymph-nodes are involved [42,44,68,69]. Clinical response did not influence the results. This lack of influence was probably due to the small number of patients who achieved clinical complete response. In the literature, some series show no correlation between tumor response to preoperative treatment and outcome [69-71], but most series suggest that there is improved outcome with increasing response to preoperative therapy [65,68,72-77]. In our series, better actuarial 5-year local control was observed among patients with primary tumor more than 6 cm from the anal verge (Table 3). The results of the Dutch CKVO 95-04 trial, which compared RT followed by surgery with only surgery, also showed better local control among patients with primary tumors located more than 5 cm from the anal verge [9]. The reason for better local control in patients with higher located tumors is probably related to the anatomic characteristics which facilitate tumor resection with wider margins. Surgical technique for tumor resection did not influence our results. Presumably, absence of a difference, including local control, is due to the fact that all patients treated with surgical resection had negative margins. Type of resection also did not influence local recurrence among the 1748 patients of the Dutch trial [9].

In our study, the acute toxicity observed was noticeable, however similar to that reported in literature. In general, the incidence of grade 3 acute toxicity during combined modality treatment ranges about 15–25% [1]. Care must be taken when CT is associated to RT during the preoperative therapy, mainly because of leucopenia that can lead patients to severe infections, septicemia and death. Whether preoperative radiochemotherapy is more toxic than only preoperative radiotherapy is an issue being addressed in the ongoing randomized EORTC trial 22921. Its preliminary results showed a greater incidence of grade 2 diarrhea in the CT group (34.3% Vs 17.3%; p < 0.005) and two patients died preoperatively from toxicity in the CT group [78]. To lessen the incidence of acute toxicity when combined radiochemotherapy is needed for pelvic tumors, intensity modulated radiation therapy (IMRT) treatment planning has been tested, because it can reduce the volume of irradiated small bowel and bone marrow [79,80]. In the future, preoperative trials with new drugs and radiotherapy with IMRT techniques will probably reduce the incidence of acute toxicity, thereby increasing the therapeutic ratio.

## Conclusion

This retrospective analysis suggests that for locally advanced rectal cancer, the preoperative combined radiochemotherapy strategy used was effective for local control. Sphincter preservation for distal rectal tumors can compromise the local control. The main adverse prognostic factors for survival and local control were fixation of the primary tumor and presence of pelvic lymph-nodes metastasis. Distant metastasis was the main pattern of failure, supporting the need of new drugs for adjuvant treatment, mainly among patients with positive lymph-nodes.
